# Sintilimab plus a bevacizumab biosimilar (IBI305) in advanced HCC with Child-Pugh A/B liver function: a real-world multicenter retrospective study

**DOI:** 10.3389/fonc.2025.1681663

**Published:** 2025-11-05

**Authors:** Yuanxu Zhang, Youhan Miao, Wei Sun, Yixing Yu, Jinjing Wang, Shuang Xiao, Shengwei Lu, Xia Wang, Yang Li, Xiucheng Pan, Weifeng Zhao

**Affiliations:** ^1^ Department of Internal Medicine, The First Affiliated Hospital of Soochow University, Suzhou, Jiangsu, China; ^2^ Department of Internal Medicine, Nantong Third People’s Hospital, Nantong, Jiangsu, China; ^3^ Department of Radiology, The First Affiliated Hospital of Soochow University, Suzhou, Jiangsu, China; ^4^ Department of Internal Medicine, The Affiliated Hospital of Xuzhou Medical University, Xuzhou, Jiangsu, China; ^5^ Department of Internal Medicine, The Affiliated Taizhou People’s Hospital of Nanjing Medical University, Taizhou School of Clinical Medicine, Nanjing Medical University, Taizhou, Jiangsu, China

**Keywords:** sintilimab, bevacizumab, Child-Pugh B, hepatocellular carcinoma, systemic treatment

## Abstract

**Background:**

Sintilimab plus a Bevacizumab biosimilar (IBI305) is an approved first-line regimen for unresectable hepatocellular carcinoma (uHCC) in China. However, data on its safety and efficacy in patients with impaired liver function remain limited. We assessed the clinical outcomes of this combination therapy in HCC patients with Child-Pugh class A (CP-A) and class B (CP-B) liver function.

**Methods:**

In this multicenter retrospective cohort study, 99 patients with advanced uHCC (73 CP-A; 26 CP-B) who received first-line Sin/Bev were included. Tumor response was assessed using modified RECIST criteria, and adverse events (AEs) were graded per CTCAE v5.0. Survival outcomes, including overall survival (OS), progression-free survival (PFS), and time to hepatic decompensation (TTD), were analyzed via Kaplan-Meier estimates and Cox proportional hazards models.

**Results:**

The objective response rates (ORR) of patients with CP-A and CP-B treated with Sin/Bev were 50.7% and 57.7%, respectively, and both could achieve good anti-tumor efficacy. CP-B had inferior survival: median OS (15 vs 22 months, p=0.044), PFS (8 vs 14 months, p=0.014), and TTD (7 vs 15 months, p<0.001). The CP-B cohort demonstrated comparable incidence rates of grade 3–4 AEs to the CP-A group (34.6% vs 34.2%). Hemorrhagic events and thrombocytopenia emerged as predominant grade 3–4 AEs in CP-B patients (15.4% for both).

**Conclusions:**

Sin/Bev demonstrated encouraging short-term anti-tumor activity in HCC of CP-A and CP-B, while survival outcomes were affected by differences in hepatic function. Although the regimen was generally well tolerated, patients with impaired liver reserve require vigilant monitoring and comprehensive supportive strategies to maximize therapeutic outcomes.

## 1 Introduction

Liver cancer is the third leading cause of cancer-related mortality globally, following lung and colorectal cancers, and ranks as the sixth most common malignancy worldwide (1). Hepatocellular carcinoma (HCC), the predominant form of primary liver cancer, is primarily associated with chronic infection with hepatitis B virus (HBV) or hepatitis C virus (HCV), as well as exposure to aflatoxins, heavy alcohol intake, obesity, diabetes, and non-alcoholic fatty liver disease (2–4). In early-stage HCC, curative interventions such as hepatic resection, liver transplantation, and local ablative therapies including percutaneous ethanol injection, radiofrequency ablation, and cryotherapy-are typically considered, contingent upon preserved hepatic function (5). However, the majority of patients present with advanced disease or impaired hepatic reserve, rendering them ineligible for surgical resection. These individuals are typically managed with systemic therapies, often in combination with locoregional approaches such as transcatheter arterial chemoembolization (TACE) or hepatic arterial infusion chemotherapy (HAIC) (6).

Recent therapeutic advances, particularly in molecular-targeted agents and immune checkpoint inhibitors, have significantly improved clinical outcomes in patients with intermediate and advanced HCC (7). Despite this progress, the pivotal clinical trials evaluating first-line systemic therapies-including the REFLECT trial (lenvatinib), the IMbrave150 trial (atezolizumab plus bevacizumab), the HIMALAYA trial (durvalumab plus tremelimumab), the CheckMate 9DW (nivolumab plus ipilimumab versus lenvatinib or sorafenib), and the CARES-310 study (camrelizumab plus apatinib)-have almost exclusively enrolled patients with preserved hepatic function, classified as Child-Pugh class A (CP-A) (8–13). In the phase II-III ORIENT-32 trial conducted in China, the combination of sintilimab (an anti-programmed death-1 [PD-1] antibody) and bevacizumab (a monoclonal antibody targeting vascular endothelial growth factor [VEGF]) significantly reduced the risk of disease progression and death in patients with unresectable HCC (14). However, only 4% of trial participants had moderately impaired hepatic function (Child-Pugh B7), leaving substantial uncertainty regarding the applicability of these findings to patients with more advanced liver dysfunction.

To address this gap, there is a pressing need for studies specifically evaluating the safety and efficacy of combination immunotherapy in patients with Child-Pugh class B (CP-B) HCC, a population that is frequently underrepresented in clinical trials yet commonly encountered in routine practice.

Parallel efforts have highlighted the albumin-bilirubin (ALBI) grade as an objective and reproducible tool for assessing liver function and prognostic stratification in patients with HCC (15). The ALBI grade correlates robustly with established hepatic scoring systems, including the Child-Pugh (CP) classification and the Model for End-Stage Liver Disease (MELD) score, and may offer enhanced discriminatory power in heterogeneous patient populations (16). Critically, recent research positions ALBI not merely as a complementary metric, but as a pivotal prognostic factor that outperforms CP classification in predicting survival outcomes (17–19). Furthermore, elevated ALBI grade independently associates with increased gastrointestinal bleeding risk following atezolizumab-bevacizumab (A+B) therapy, underscoring its clinical utility for risk stratification during systemic treatment regimens (17).

In this multicenter retrospective cohort study, we evaluated the clinical outcomes of patients with advanced HCC and CP-A or CP-B hepatic function who received sintilimab in combination with bevacizumab as first-line systemic therapy in real-world settings across four institutions in China. Beyond the Child-Pugh classification, we further sought to characterize liver function using the albumin-bilirubin (ALBI) grade, with a particular focus on exploring whether on-treatment ALBI dynamics could serve as an early indicator for long-term survival.

## 2 Methods

### 2.1 Patients

We conducted a multicenter, retrospective analysis of patients seen at The First Affiliated Hospital of Soochow University, Taizhou People’s Hospital, Nantong Third People’s Hospital, and The Affiliated Hospital of Xuzhou Medical University.

Patients who had a prior diagnosis of HCC classified as Barcelona Clinic Liver Cancer (BCLC) stage B or C, and CP-A or B, and received sintilimab plus bevacizumab biosimilar (IBI305) as first-line systemic therapy were included between August 2020 and December 2024. The diagnosis of HCC was confirmed by either histopathological examination or non-invasive assessment, in accordance with criteria established by the American Association for the Study of Liver Diseases (AASLD) for patients with cirrhosis. Specifically, for non-invasive diagnosis, typical imaging findings on dynamic contrast-enhanced computed tomography (CT) or magnetic resonance imaging (MRI) were required, in combination with elevated serum alpha-fetoprotein (AFP) levels, in line with AASLD guidelines.

Eligibility required patients ≥18 years with radiologically measurable disease, not amenable to curative surgical resection or locoregional therapies, such as radiofrequency ablation, TACE, or HAIC, or to have experienced disease progression following such treatments. To avoid potential confounding effects, a minimum interval of 6 weeks was required between the last locoregional therapies and the initiation of sintilimab plus bevacizumab. Patients were excluded if they had Child-Pugh class C cirrhosis, radiologically unmeasurable intrahepatic lesions, confirmed non-HCC malignancies, incomplete baseline or follow-up data, or loss of follow-up.

### 2.2 Treatment and assessment

The treatment regimen followed the standard institutional protocol. Dose modifications, including temporary interruption or dose reduction, were implemented at the discretion of the attending physicians, based on each patient’s clinical status, tolerance, and adverse event (AE) profile. Treatment with sintilimab and bevacizumab was continued until the onset of intolerable toxicity or radiological confirmation of disease progression.

Tumor assessment was conducted using dynamic contrast-enhanced CT or MRI. Baseline imaging was obtained prior to treatment initiation, and follow-up scans were performed every 6–8 weeks in accordance with institutional guidelines. Tumor response was evaluated using the modified Response Evaluation Criteria in Solid Tumours (mRECIST), which involves measuring the diameters of target lesions to determine whether the response status corresponds to complete response, partial response, stable disease, or progressive disease. Treatment-emergent adverse events were graded using the National Cancer Institute Common Terminology Criteria for Adverse Events version 5.0.

### 2.3 Statistical analyses

Patient demographics and clinical characteristics were stratified by Child-Pugh class for comparative analysis. Categorical variables were expressed as frequencies and percentages and analyzed using either the Chi-square test or Fisher’s exact test. Continuous variables were reported as mean and standard deviation (SD).

The Kaplan-Meier method was used to calculate median overall survival (OS), progression-free survival (PFS), and time to hepatic decompensation (TTD). OS was defined as the interval from initiation of sintilimab plus bevacizumab to death from any cause. Patients who were alive at the time of data cutoff (December 30, 2024) were censored on that date. PFS was defined as the time from treatment initiation to radiologically confirmed progression based on mRECIST or death from any cause, with censoring at the last follow-up for those without an event. TTD was defined as the interval from the first dose of therapy to the occurrence of either: (a) laboratory-confirmed decline in hepatic synthetic function (e.g. serum bilirubin elevation), or (b) clinical signs of portal hypertension, such as hepatic encephalopathy, ascites requiring intervention, or acute variceal bleeding. Safety outcomes in the CP-B group were compared with those in the CP-A group. Univariate and multivariate analyses were conducted using Cox proportional hazards regression to evaluate prognostic factors for OS and PFS. Missing data were handled using a complete-case analysis approach, excluding cases with unavailable key variables from the relevant statistical tests. A two-sided p-value of <0.05 was considered statistically significant. All statistical analyses were performed using the Statistical Package for the Social Sciences (SPSS) version 27.0 (IBM Corp, Armonk, NY, USA).

## 3 Results

### 3.1 Patients characteristics

**Figure 1:**
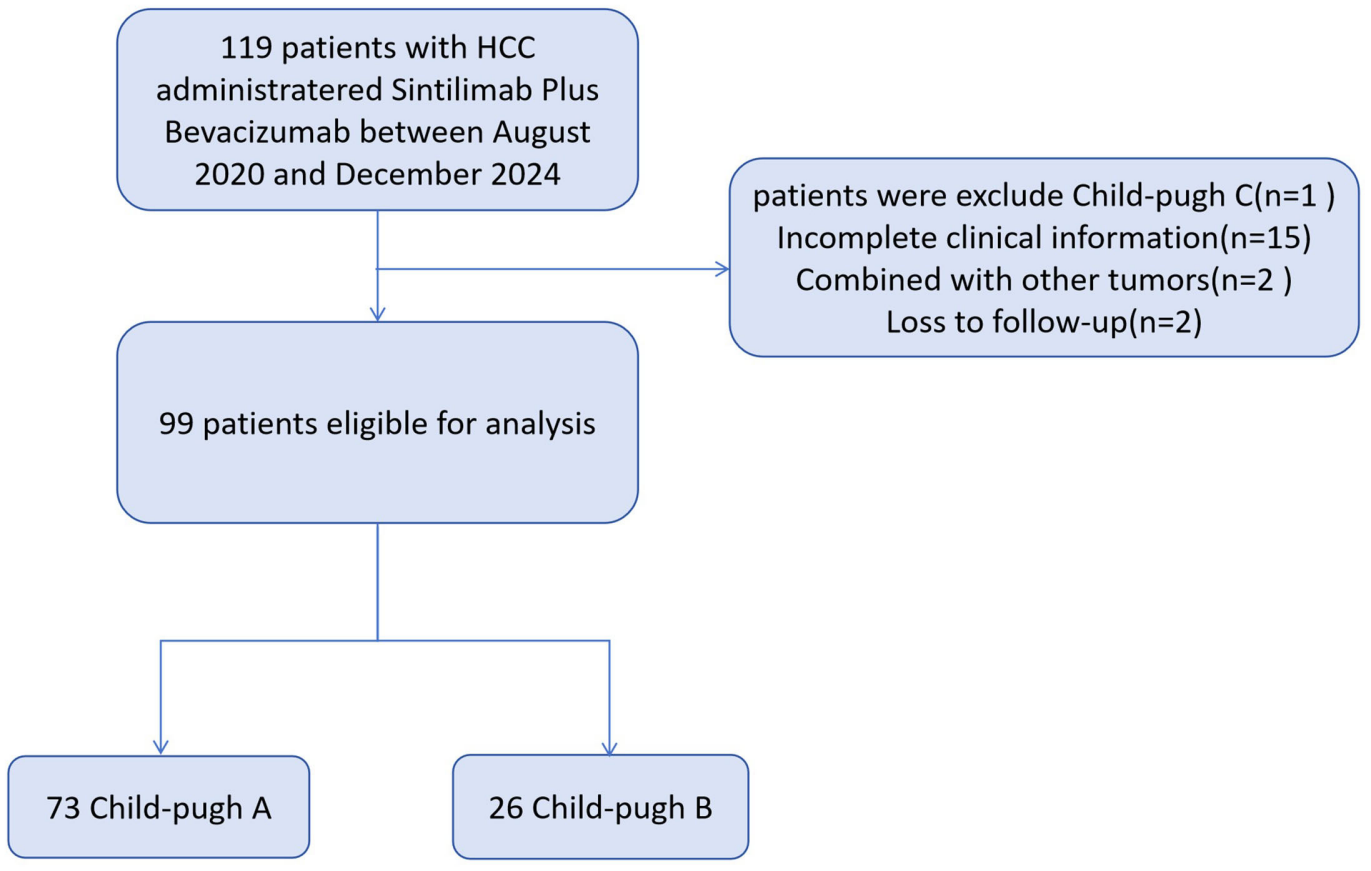
Flowchart for patient selection.

Baseline characteristics are summarized in **Table 1**. From August 2020 to December 2024, a total of 99 patients with unresectable HCC were included (73 Child-Pugh A; 26 Child-Pugh B; **Figure 1**). The median age was 61 years in both groups, and most patients were male, the proportion of males was 72.2% in Child-Pugh Group A and 76.9% in Group B. Hepatitis B virus was the predominant etiology, present in 79.5% of Child-Pugh A and 65.4% of Child-Pugh B patients. Multifocal tumors (≥3 lesions) were more frequent in CP-B (76.9% vs 67.1%). The CP-A cohort had a higher rate of prior local treatment than the CP-B cohort (61.6% vs 53.8%), with TACE being the most common. Extrahepatic metastasis occurred in 23.1% of CP-B patients, mainly adrenal involvement. In CP-A, lymph node metastasis was most common, followed by lung and bone.

**Table 1:** Patient baseline characteristics, and prior local therapies.

Characteristics	Child–Pugh A(n=73)	Child–Pugh B(n=26)	p-value
Age at diagnosis (years), median (IQR)	61 (32, 82)	61 (47,80)	0.558
<65	44 (60.3%)	17 (65.4%)	
≥65	29 (39.7%)	9 (34.6%)	
Sex			0.567
Female	21 (28.8%)	6 (23.1%)	
Male	52 (72.2%)	20 (76.9%)	
Etiology			
Hepatitis B			0.151
No	15 (20.5%)	9 (34.6%)	
Yes	58 (79.5%)	17 (65.4%)	
Hepatitis C			0.582
No	68 (93.2%)	25 (96.2%)	
Yes	5 (6.8%)	1 (3.8%)	
Alcohol			0.067
No	73 (100.0%)	24 (92.3%)	
Yes	0 (0.0%)	2 (7.7%)	
MASH			0.458
No	72 (98.6%)	25(96.2%)	
Yes	1 (1.4%)	1 (3.8%)	
Schistosomiasis			0.263
No	73 (100.0%)	25 (96.2%)	
Yes	0 (0.0%)	1 (3.8%)	
Primary biliary cirrhosis			0.263
No	73 (100.0%)	25 (96.2%)	
Yes	0 (0.0%)	1 (3.8%)	
Unknown	12 (16.4%)	5 (19.2%)	0.983
Liver cirrhosis	47 (64.4%)	19 (73.1%)	0.419
Tumor number			0.350
<3	24 (32.9%)	6 (23.1%)	
≥3	49 (67.1%)	20 (76.9%)	
Tumor size, cm			0.079
<5	31 (42.5%)	6 (23.1%)	
≥5	42 (57.5%)	20 (76.9%)	
AFP, ng/mL			0.153
<400	48 (65.8%)	21 (80.8%)	
≥400	25 (34.2%)	5 (19.2%)	
DCP, ug/L			
<900	44 (60.3%)	14 (53.8%)	0.568
≥900	29 (39.7%)	12 (46.2%)	
BCLC stage			0.038
B	20 (27.4%)	2 (7.7%)	
C	53 (72.6%)	24 (92.3%)	
ALBI grade			0.004
1	27 (37.0%)	3 (11.5%)	
2	46 (63.0%)	20 (76.9%)	
3	0 (0.0%)	3 (11.5%)	
ECOG PS			0.017
0	35 (47.9%)	8 (30.8%)	
1	31 (42.5%)	10 (38.5%)	
2	7 (9.6%)	8 (30.8%)	
Vascular invasion	20 (27.4%)	17 (65.4%)	<0.01
Varices	9(12.3%)	7(26.9%)	0.083
Extrahepatic spread	18 (24.7%)	6 (23.1%)	0.872
Lymph node	15 (20.5%)	3 (11.5%)	
Lung	2 (2.74%)	0 (0.0%)	
Bone	2 (2.74%)	0 (0.0%)	
Peritoneum	1 (1.37%)	1 (3.85%)	
adrenal gland	0 (0.0%)	4 (15.4%)	
Ascites			<0.01
Absent	58 (79.5%)	4 (15.4%)	
Slight	15 (20.5%)	7 (26.9%)	
Moderate	0 (0.0%)	15 (57.7%)	
Encephalopathy			0.017
None	73 (100%)	23 (88.5%)	
Grade 1-2	0 (0%)	3 (11.5%)	
Prior treatment	51 (69.9%)	21 (80.8%)	0.284
Surgery	21 (28.8%)	10 (38.5%)	
TACE	37 (50.7%)	16 (61.5%)	
HAIC	18 (24.7%)	10 (38.5%)	
RFA	7 (9.6%)	2 (7.7%)	

AFP, Alpha-fetoprotein; ALBI, Albumin–bilirubin; BCLC, Barcelona clinic liver cancer; ECOG, Eastern cooperative oncology group; RFA, Radiofrequency ablation; TACE, Transcatheter arterial chemoembolization.

The CP-B cohort showed more severe disease features than CP-A. They had higher proportions of BCLC stage C (92.3% vs 72.6%; p=0.038), ALBI grade 3 (11.5% vs 0.0%; p=0.004), ECOG PS 2 (30.8% vs 9.6%; p=0.017), and vascular invasion (65.4% vs 27.4%; p<0.01). Moderate ascites (57.7% vs 0.0%; p<0.01) and hepatic encephalopathy (11.5% vs 0.0%; p=0.017) were also significantly more frequent in CP-B.

### 3.2 Treatment outcomes

Treatment responses are summarized in **Table 2**. Both Child-Pugh cohorts demonstrated robust antitumor activity based on mRECIST assessments, with comparable objective response rates (ORR) observed between CP-A and CP-B subgroups (50.7% vs 57.7%). In the Child-Pugh A group (n=73), a complete response (CR) was achieved in 4 patients (5.5%), and a partial response (PR) was observed in 33 patients (45.2%). In the CP-B subgroup, 1 (3.8%) and 14 (53.8%) patients had CR and PR, respectively. Among CP-B8–9 patients (n=11), an objective response rate (ORR) of 63.6% (7/11) was achieved. This robust efficacy underscores the therapeutic potential of this regimen in advanced liver dysfunction and addresses a pivotal unmet clinical need in this marginalized subgroup. The CP-A cohort demonstrated a higher disease control rate (DCR, CR+PR+SD) compared with the CP-B group (83.6% vs 69.2%).

**Table 2:** Tumor responses as per mRECIST.

	Child–Pugh A5 (n=43)	Child–Pugh A6 (n=30)	Child–Pugh A (n=73)	Child–Pugh B7 (n=15)	Child–Pugh B8 (n=5)	Child–Pugh B9 (n=6)	Child–Pugh B (n=26)	Total (n=99)
Best response								
CR	3	1	4	1	0	0	1	5
PR	21	12	33	7	3	4	14	46
SD	13	11	24	2	1	0	3	27
PD	6	6	12	5	1	2	8	21
ORR	55.8%	43.3%	50.7%	53.3%	60.0%	66.7%	57.7%	51.5%
DCR	86.0%	80.0%	83.6%	66.7%	80.0%	66.7%	69.2%	78.8%

CR, Complete response; PR, Partial response; SD, Stable disease; PD, Progressive disease; ORR, Objective response rate; DCR, Disease controlrate.

**Figure 2:**
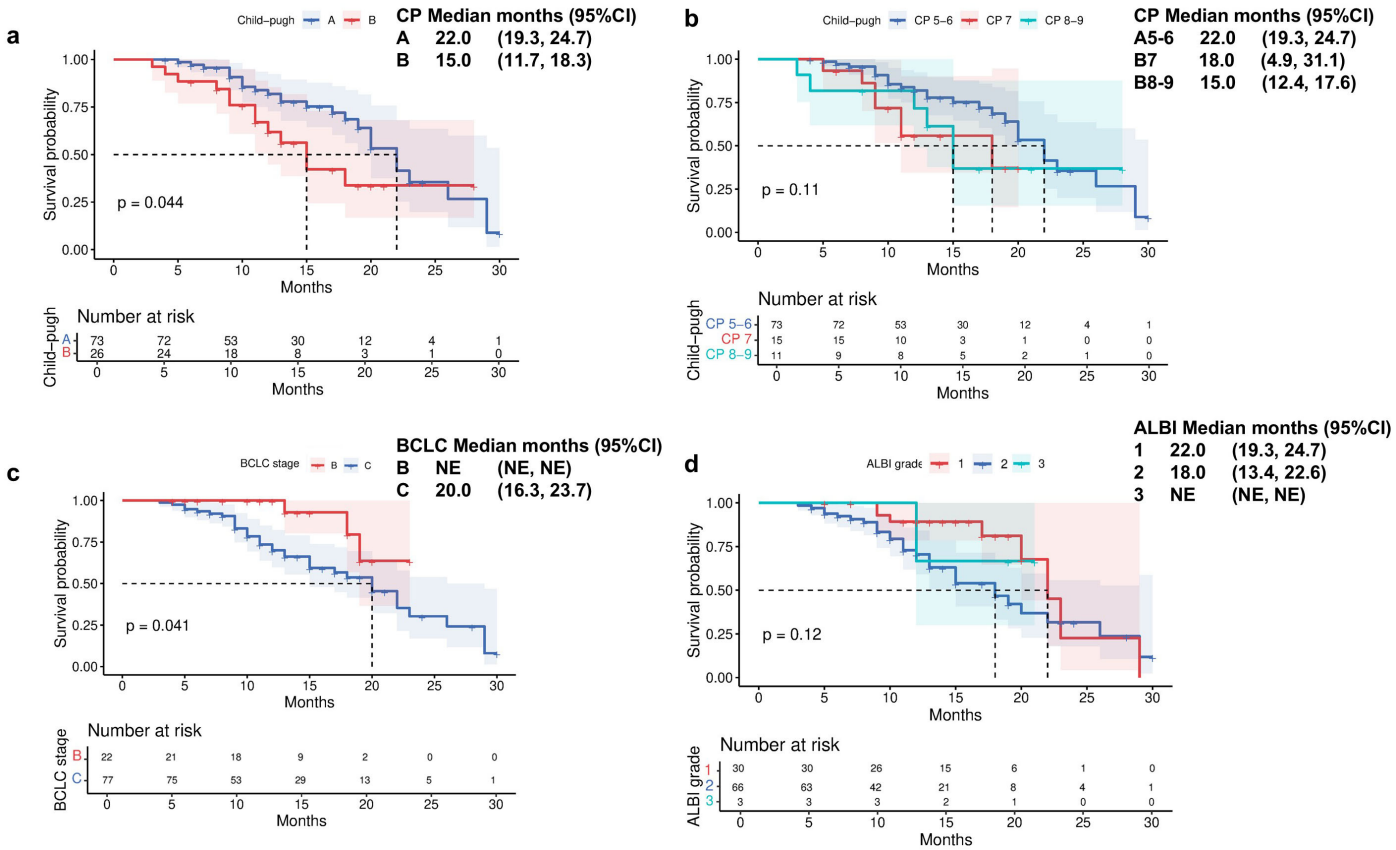
Overall survival by **(a)** Child-Pugh liver status (A vs B), **(b)** Child-Pugh liver status (A5/6 vs B7 vs B8/9), **(c)** Barcelona Clinic Liver Cancer stage (B vs C), and **(d)** ALBI grade liver function.

**Figure 3:**
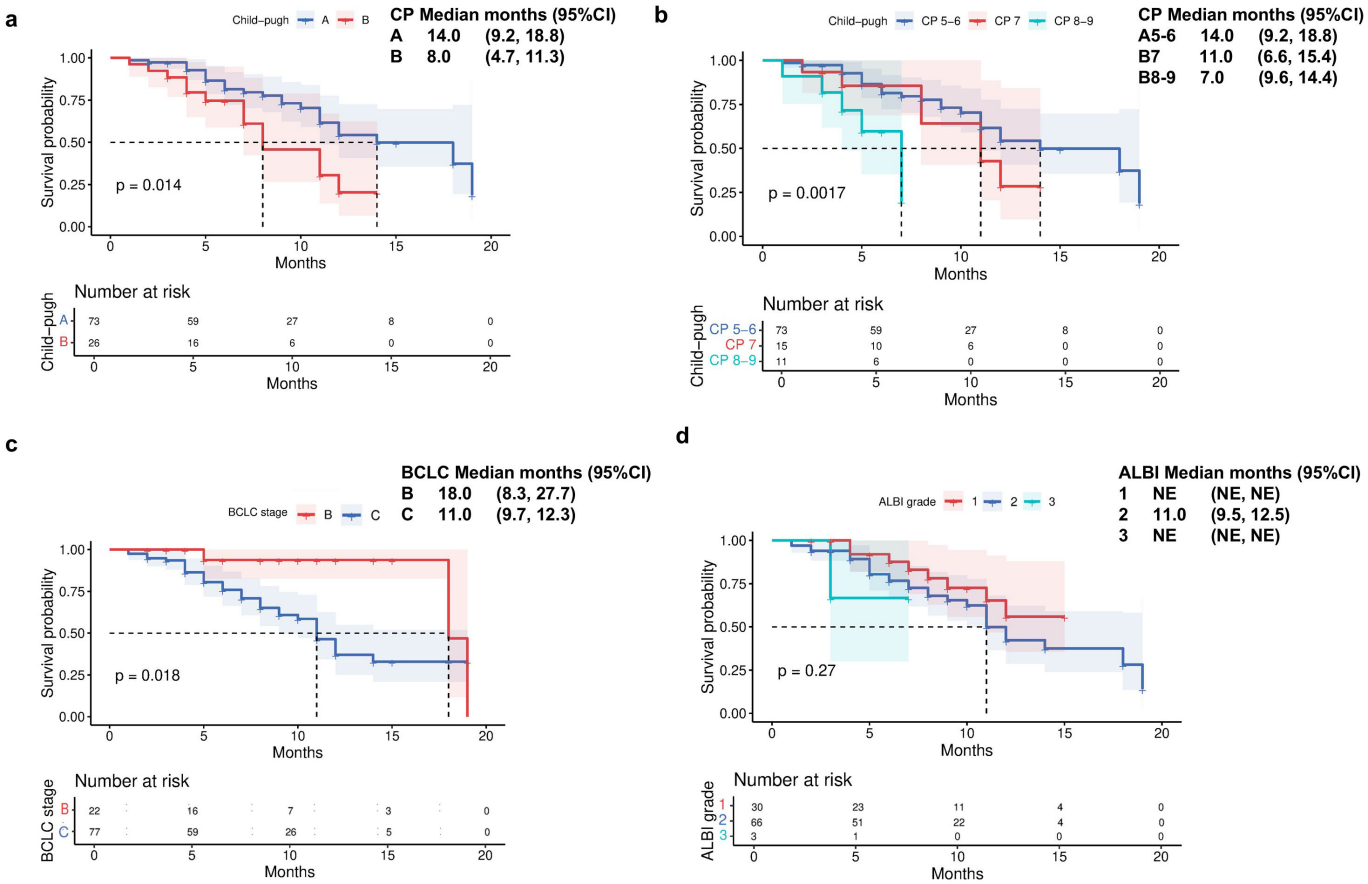
Progression-free survival by **(a)** Child-Pugh liver status (A vs B), **(b)** Child-Pugh liver status (A5/6 vs B7 vs B8/9), **(c)** Barcelona Clinic Liver Cancer stage (B vs C), and **(d)** ALBI grade liver function.

Patients with CP-A liver function demonstrated a significantly longer median OS of 22 months (95% CI 19.3-24.7) compared to 15 months (95% CI 11.7-18.3) in those with CP-B liver function (**Figure 2a**). Further stratification of CP-B patients revealed distinct survival patterns: those with CP-B7 achieved an mOS of 18 months (95% CI 4.9-31.1), whereas patients with more advanced CP-B8–9 liver dysfunction showed a reduced mOS of 15 months (95% CI 12.4–17.6; **Figure 2b**). Consistent with liver function analysis, the Barcelona clinical hepatocellular carcinoma (BCLC) stage also affected the survival indicators OS (P = 0.041; **Figure 2c**). When evaluating survival based on ALBI score, patients with ALBI grade 1 had an mOS of 22 months (95% CI 19.3-24.7), while those with grade 2 had an mOS of 18 months (95% CI 13.4-22.6; **Figure 2d**). Analysis of PFS according to baseline liver function revealed significant disparities between CP classes. Patients with CP-A exhibited a median PFS of 14 months (95% CI 9.2-18.8), substantially longer than the 8-month mPFS in CP-B patients (95% CI 4.7-11.3; **Figure 3a**; p=0.014). Subgroup analysis of CP-B patients demonstrated a gradational decline: CP-B7 patients maintained an mPFS of 11 months (95% CI 6.6-15.4), whereas those with CP-B8–9 showed faster progression with an mPFS of 7 months (95% CI 5.3-8.7; **Figure 3b**). Consistent with liver function stratification, analysis by BCLC stage revealed significant differences (p=0.018): BCLC-B patients achieved an mPFS of 18 months (95% CI 8.3-27.7), compared with 11 months (95% CI 9.7-12.3) for BCLC-C patients (**Figure 3c**). Patients with ALBI grade 2 had an mPFS of 11 months (95% CI 9.5-12.5; **Figure 3d**).

**Figure 4:**
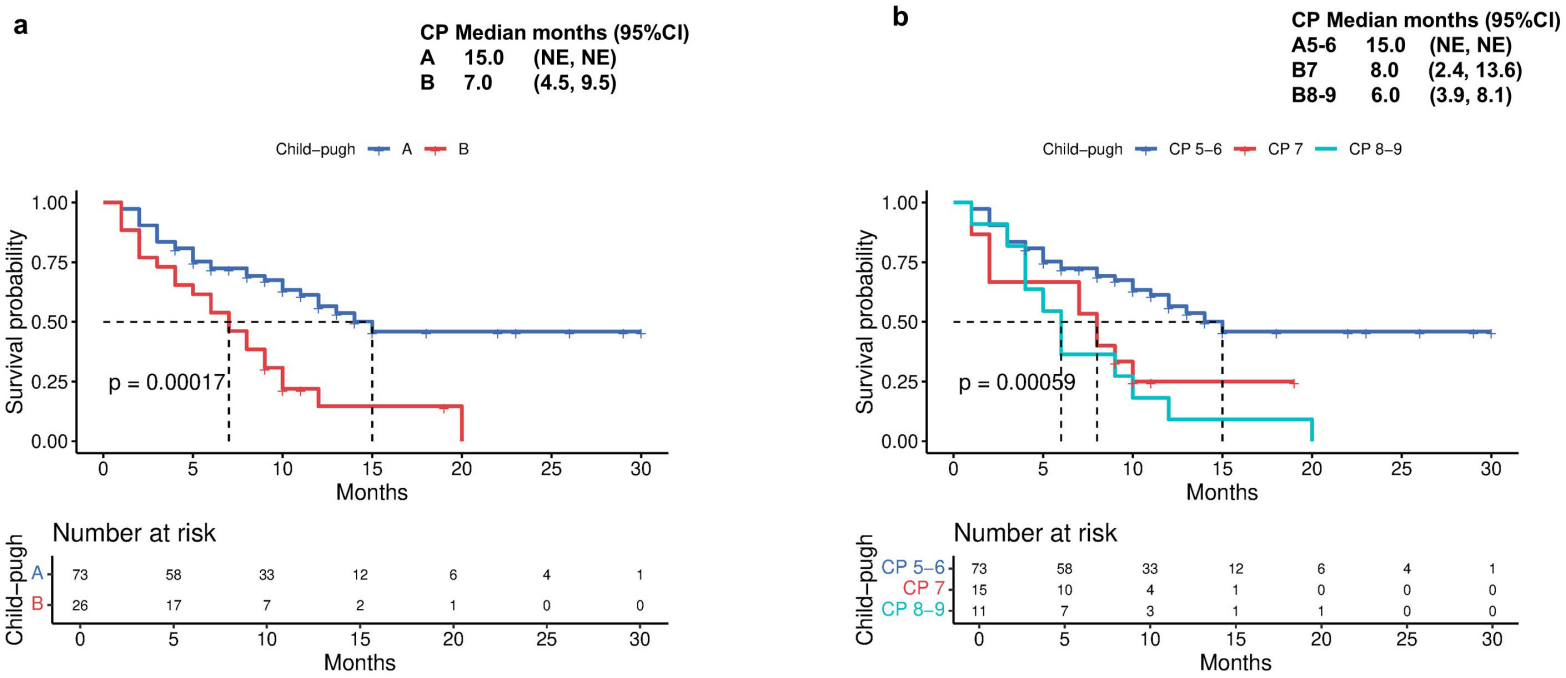
Time to decompensation (TTD) by **(a)** Child-Pugh liver status (A vs B) and **(b)** Child-Pugh liver status (A5/6 vs B7 vs B8/9).

The median TTD was 15 months for CP-A patients, compared with 7 months (95% CI 4.5-9.5) for CP-B patients (p<0.001; **Figure 4a**). Subgroup analysis within CP-B showed that CP-B7 patients had a median TTD of 8 months (95% CI 2.4-13.6), while CP-B 8–9 patients had a reduced median of 6 months (95% CI 3.9-8.1; **Figure 4b**).

**Figure 5:**
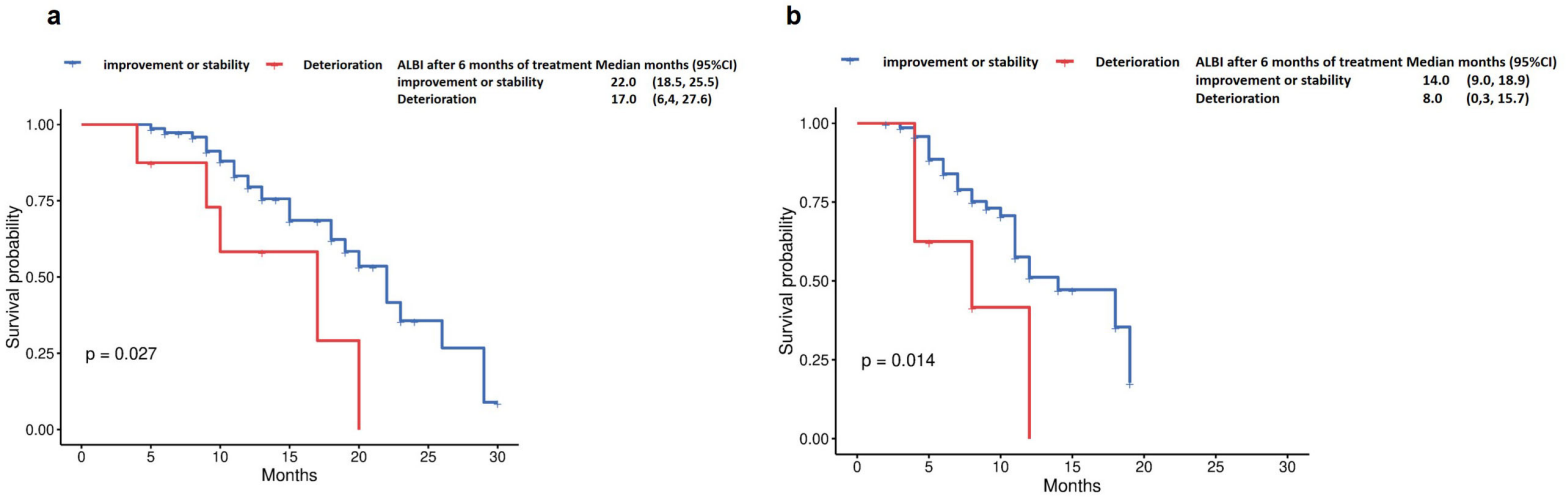
Overall survival by **(a)** ALBI after 6 months of treatment (Deterioration vs improvement or stability), and Progression-free survival by **(b)** ALBI after 6 months of treatment (Deterioration vs improvement or stability).

Patients with improved ALBI grade at 6 months (n=84) demonstrated significantly longer OS compared to those with deteriorated ALBI (22 months vs 17 months, p=0.027, **Figure 5a**). Similarly, progression-free survival was markedly prolonged in the ALBI-improvement cohort (14 months vs 8 months, p=0.014, **Figure 5b**).

**Figure 6:**
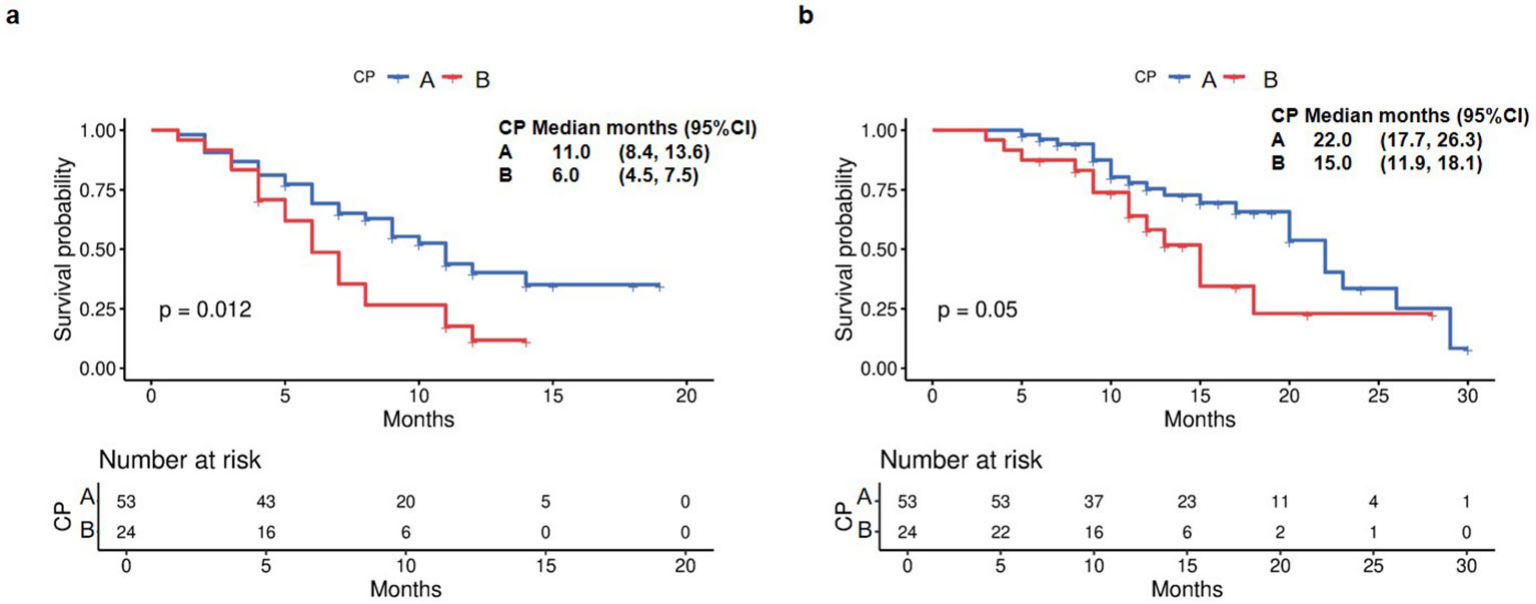
Kaplan Meier survival curves of PFS and OS in the BCLC-C subgroup. **(a)** PFS in the BCLC-C subgroup. **(b)** OS in the BCLC-C subgroup.

To account for the baseline imbalance in tumor stage between CP-A and CP-B groups, we performed subgroup analyses restricted to patients with BCLC-C stage (n=77). This analysis revealed that median PFS was 11 months for CP-A versus 6 months for CP-B (p=0.012, **Figure 6a**). Median OS was 22 months for CP-A patients versus 15 months for CP-B patients (p=0.05, **Figure 6b**).

### 3.3 Univariate and multivariate analysis for factors predictive of survival

**Figure 7:**
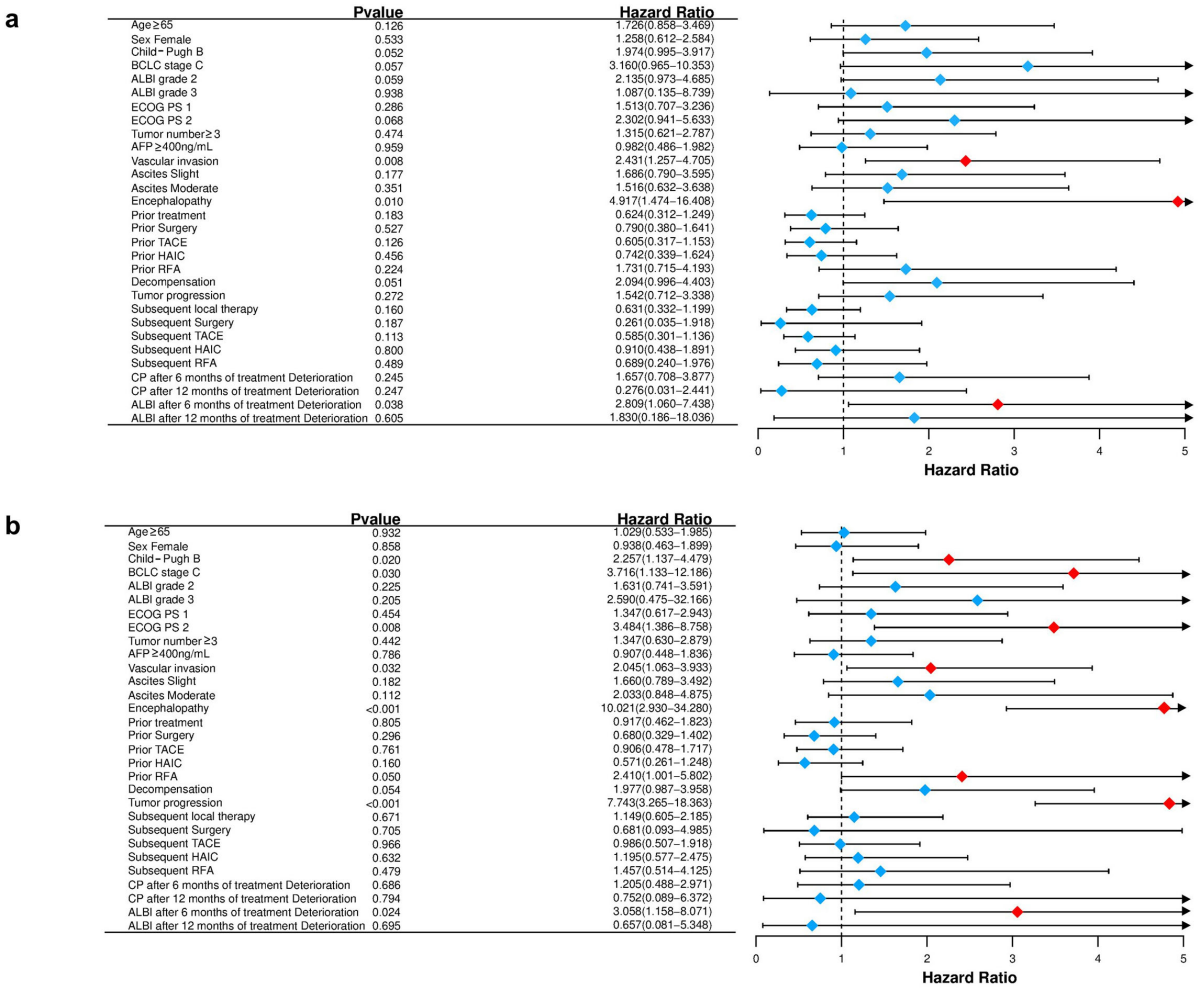
Forest plot for overall survival **(a)** and progression-free survival **(b)** of the matched cohorts of patients.

In univariate analysis (**Table 3**; **Figure 7a**), several variables demonstrated prognostic significance for overall survival (OS). A higher CP score was associated with worse OS. Specifically, patients with CP-B had a hazard ratio (HR) of 1.974 (95% CI 0.995-3.917; p=0.052) compared with CP-A, indicating a potential negative impact. BCLC stage C showed a trend toward unfavorable prognosis versus stage B (HR 3.160, 95% CI 0.965-10.353; p=0.057). Eastern Cooperative Oncology Group (ECOG) performance status (PS) was a critical factor, with PS 2 associated with poorer OS compared to PS 0 (HR 2.302, 95% CI 0.941-5.633; p=0.068). Vascular invasion was significantly associated with reduced OS (HR 2.431, 95% CI 1.257-4.705; p=0.008), as was the presence of encephalopathy (HR 4.917, 95% CI 1.474-16.408; p=0.010).

**Table 3:** Univariate analysis for factors predictive of progression free survival and overall survival.

Independent variables	Level	PFS		OS	
		HR (95% CI)	P-value	HR (95% CI)	P-value
Age	<65	Reference	–	Reference	–
	≥65	1.029 (0.533,1.985)	0.932	1.726 (0.858,3.469)	0.126
Sex	Male	Reference	–	Reference	–
	Female	0.938 (0.463,1.899)	0.858	1.258 (0.612,2.584)	0.533
Child–Pugh	CP-A	Reference	–	Reference	–
	CP-B	2.257 (1.137,4.479)	**0.020**	1.974 (0.995,3.917)	**0.052**
	CP-A 5-6				
	CP-B 7	1.582 (0.677,3.696)	0.289	2.231 (0.939,5.299)	0.069
	CP-B 8-9	4.865 (1.839,12.868)	**0.001**	1.748 (0.710,4.304)	0.224
BCLC stage	B	Reference	–	Reference	–
	C	3.716 (1.133,12.186)	**0.030**	3.160 (0.965,10.353)	0.057
ALBI grade	1	Reference	–	Reference	–
	2	1.631 (0.741,3.591)	0.225	2.135 (0.973,4.685)	0.059
	3	2.590 (0.475,32.166)	0.205	1.087 (0.135,8.739)	0.938
ECOG PS	0	Reference	–	Reference	–
	1	1.347 (0.617,2.943)	0.454	1.513 (0.707,3.236)	0.286
	2	3.484 (1.386,8.758)	**0.008**	2.302 (0.941,5.633)	0.068
Tumor number	<3	Reference	–	Reference	–
	≥3	1.347 (0.630,2.879)	0.442	1.315 (0.621,2.787)	0.474
Tumor size, cm	<5	Reference	–	Reference	–
	≥5	1.579 (0.909,2.743)	0.105	1.330 (0.684,2.587)	0.400
AFP, ng/mL	<400	Reference	–	Reference	–
	≥400	0.907 (0.448,1.836)	0.786	0.982 (0.486,1.982)	0.959
Liver cirrhosis	No	Reference	–	Reference	–
	Yes	1.000 (0.492,2.035)	1.000	0.718 (0.344,1.501)	0.379
Vascular invasion	No	Reference	–	Reference	–
	Yes	2.045 (1.063,3.933)	**0.032**	2.431 (1.257,4.705)	**0.008**
Ascites	Absent	Reference	–	Reference	–
	Slight	1.660 (0.789,3.492)	0.182	1.686 (0.790,3.595)	0.177
	Moderate	2.033 (0.848,4.875)	0.112	1.516 (0.632,3.638)	0.351
Encephalopathy	None	Reference	–	Reference	–
	Grade 1-2	10.021 (2.930,34.280)	**<0.001**	4.917 (1.474,16.408)	**0.010**
Prior treatment	YES/NO	0.917 (0.462,1.823)	0.805	0.624 (0.312,1.249)	0.183
Prior Surgery	YES/NO	0.680 (0.329,1.402)	0.296	0.790 (0.380,1.641)	0.527
Prior TACE	YES/NO	0.906 (0.478,1.717)	0.761	0.605 (0.317,1.153)	0.126
Prior HAIC	YES/NO	0.571 (0.261,1.248)	0.160	0.742 (0.339,1.624)	0.456
Prior RFA	YES/NO	2.410 (1.001,5.802)	0.050	1.731 (0.715,4.193)	0.224
Decompensation	YES/NO	1.977 (0.987,3.958)	0.054	2.094 (0.996,4.403)	0.051
Tumor progression	YES/NO	7.743 (3.265,18.363)	**<0.001**	1.542 (0.712,3.338)	0.272
Subsequent local therapy	YES/NO	1.149 (0.605,2.185)	0.671	0.631 (0.332,1.199)	0.160
Subsequent Surgery	YES/NO	0.681 (0.093,4.985)	0.705	0.261 (0.035,1.918)	0.187
Subsequent TACE	YES/NO	0.986 (0.507,1.918)	0.966	0.585 (0.301,1.136)	0.113
Subsequent HAIC	YES/NO	1.195 (0.577,2.475)	0.632	0.910 (0.438,1.891)	0.800
Subsequent RFA	YES/NO	1.457 (0.514,4.125)	0.479	0.689 (0.240,1.976)	0.489
CP after 6 months of treatment	Deterioration/improvement or stability	1.205 (0.488,2.971)	0.686	1.657 (0.708,3.877)	0.245
CP after 12 months of treatment	Deterioration/improvement or stability	0.752 (0.089,6.372)	0.794	0.276 (0.031,2.441)	0.247
ALBI after 6 months of treatment	Deterioration/improvement or stability	3.058 (1.158,8.071)	**0.024**	2.809 (1.060,7.438)	**0.038**
ALBI after 12 months of treatment	Deterioration/improvement or stability	0.657 (0.081,5.348)	0.695	1.830 (0.186,18.036)	0.605

Child-Pugh and ALBI scores were evaluable in 84/99 patients at 6 months and in 29/99 patients at 12 months after treatment. Bold statistically significant (p<0.05).

For PFS (**Figure 7b**), univariate analysis revealed that CP-B patients had a higher risk of progression (HR 2.257, 95% CI 1.137-4.479; p=0.020). Higher ALBI grades showed a trend toward negative prognosis, with ALBI grade 3 associated with HR 2.590 (95% CI 0.475-32.166; p=0.205). ECOG PS 2 was significantly associated with poorer PFS (HR 3.484, 95% CI 1.386-8.758; p=0.008). Tumor progression was a strong negative predictor (HR 7.743, 95% CI 3.265-18.363; p<0.001). Prior RFA was associated with a favorable trend (HR 2.410, 95% CI 1.001-5.802; p=0.050).

In multivariate analysis for OS (**Table 4**), CP-B status remained an independent predictor of reduced survival (HR 2.540, 95% CI 1.016-6.345; p=0.046). Deterioration in ALBI grade after 6 months of treatment was also an independent predictor of worse OS (HR 3.204, 95% CI 1.056-9.723; p=0.040). For PFS, CP-B showed a non-significant trend (HR 1.857, 95% CI 0.783-4.402; p=0.160), while ALBI grade deterioration after 6 months remained a significant negative prognostic factor (HR 3.353, 95% CI 1.083–10.384; p=0.036).

**Table 4:** Multivariate analysis for factors predictive of progression free survival and overall survival.

Variables	Comparison	PFS		OS	
		HR (95% CI)	P-value	HR (95% CI)	P-value
Child–Pugh	CP-B vs CP-A	1.857 (0.783,4.402)	0.160	2.540 (1.016,6.345)	**0.046**
BCLC	C vs B	3.468 (0.819,14.689)	0.091	1.616 (0.455,5.734)	0.458
ECOG PS	0	–	–	–	–
	1	0.809 (0.294,2.222)	0.681	–	–
	2	1.921 (0.563,6.551)	0.297	–	–
Vascular invasion	YES vs NO	0.480 (0.167,1.377)	0.172	1.696 (0.720,3.994)	0.227
Encephalopathy	YES vs NO	4.567 (0.760,27.424)	0.097	1.465 (0.291,7.375)	0.644
Decompensation	YES vs NO	1.292 (0.499,3.345)	0.598	–	–
ALBI after 6 months of treatment	Deterioration/improvement or stability	3.353 (1.083,10.384)	**0.036**	3.204 (1.056,9.723)	**0.040**

CI, Confidence interval. Bold statistically significant (p<0.05).

### 3.4 Safety

The majority of patients experienced adverse events (AEs), with a comparable incidence between CP-A (90.4%, 66/73) and CP-B (96.2%, 25/26) groups (p=0.615; **Table 5**).

**Table 5:** Adverse events.

	All Grade			Grade3~5		
	CP-A(n=73)	CP-B(n=26)	P-value	CP-A(n=73)	CP-B(n=26)	P-value
Total	66(90.4%)	25(96.2%)	0.615	25(34.2%)	9(34.6%)	0.973
Fatigue	13(17.8%)	7(26.9%)	0.320	1(1.4%)	0(0.0%)	1.000
Rash	1(1.4%)	2(7.7%)	0.168	-	-	-
Diarrhea	2(2.7%)	1(3.8%)	1.000	-	-	-
Hypertension	13(17.8%)	5(19.2%)	1.000	-	-	-
Bleeding	8(11.0%)	7(26.9%)	0.103	4(5.5%)	4(15.4%)	0.241
Hypothyroidism	4(5.5%)	2(7.7%)	1.000	-	-	-
Fever	4(5.5%)	1(3.8%)	1.000	-	-	-
Lung infection	3(4.1%)	1(3.8%)	1.000	3(4.1%)	1(3.8%)	1.000
Immune-related AE	2(2.7%)	0(0.0%)	1.000	2(2.7%)	0(0.0%)	1.000
Neutropenia	18(24.7%)	7(26.9%)	0.819	4(5.5%)	2(7.7%)	1.000
Anemia	11(15.1%)	5(19.2%)	0.853	2(2.7%)	1(3.8%)	1.000
Thrombocytopenia	29(39.7%)	13(50.0%)	0.363	8(11.0%)	4(15.4%)	0.807
Elevated AST	9(12.3%)	6(23.1%)	0.320	1(1.4%)	0(0.0%)	1.000
Elevated ALT	6(8.2%)	3(11.5%)	0.914	-	-	-
Hyperbilirubinemia	8(11.0%)	3(11.5%)	1.000	2(2.7%)	0(0.0%)	1.000
Hypoalbuminemia	44(60.3%)	22(84.6%)	**0.024**	1(1.4%)	0(0.0%)	1.000
Creatinine increased	29(39.7%)	9(34.6%)	0.645	3(4.1%)	0(0.0%)	0.564
Proteinuria	8(11.0%)	2(7.7%)	0.924	1(1.4%)	0(0.0%)	1.000

Bold statistically significant (p<0.05).

In the CP-A group (n=73), the most frequent adverse events (AEs) of any grade were hypoalbuminaemia (n=44, 60.3%), thrombocytopenia (n=29, 39.7%), and increased creatinine (n=29, 39.7%). In the CP-B group (n=26), hypoalbuminemia (22 cases, 84.6%) and thrombocytopenia (n=13, 50.0%) were most frequently observed. Hypoalbuminemia showed a significant difference between the two groups (60.3% vs 84.6%, p=0.024). Notably, any-grade bleeding was more frequent in CP-B patients (26.9% vs 11.0%; p=0.103), though this did not reach statistical significance.

The incidence of grade 3–5 AEs was similar across groups, with 34.2% (25/73) in CP-A and 34.6% (9/26) in CP-B (p=0.973). The most common included thrombocytopenia (n=12, 13.2%) and bleeding (n=8, 8.8%). One patient died due to gastrointestinal bleeding.

Ten patients (10.1%) discontinued treatment due to AEs, primarily from bleeding (n=6, 6.06%), immune-related pneumonitis (n=1, 1.01%), diarrhea (n=1, 1.01%), fatigue (n=1, 1.01%), and proteinuria (n=1, 1.01%).

## 4 Discussion

In this retrospective study, we evaluated the efficacy and safety of sintilimab plus bevacizumab in patients with unresectable HCC and impaired liver function (Child-Pugh B) across four tertiary centers. Compared with the ORIENT-32 trial, this study demonstrated superior outcomes with sintilimab-bevacizumab in HCC patients, showing a median overall survival of 20 months and objective response rate of 51.5% (14). This enhanced efficacy may be attributed to rigorous liver function management during treatment and combined local therapies administered to most patients (53/99; including TACE [n=45], HAIC [n=27], radiofrequency ablation [n=10], and surgery [n=6]). Post-progression treatment may have also affected survival outcomes. After disease progression, 47.6% of patients continued sintilimab plus bevacizumab, including four who received additional locoregional therapy, while others switched to tislelizumab plus donafenib (14.2%), lenvatinib (14.2%), or apatinib (19.0%). Notably, ORIENT-32 was conducted during the COVID-19 pandemic, where treatment delays and deferred tumor assessments may have compromised response evaluation and attenuated the observed response rate.

Unlike randomized controlled trials (e.g., ORIENT-32) and a Chinese retrospective study that primarily enrolled CP-A patients, our real-world analysis encompassed both CP-A and CP-B populations across four tertiary centers (14, 20). This study demonstrates that sintilimab-bevacizumab achieved clinically meaningful outcomes in the CP-B HCC, with an objective response rate (ORR) of 57.7% and median overall survival (OS) of 15 months. In the CP-A group, the ORR (50.7%) was comparable to that of CP-B, and the OS was longer (22 months).

The shorter survival observed in CP-B patients despite a similar response rate may be explained by impaired hepatic reserve. This conclusion is further reinforced by our subgroup analysis, which confirmed the survival disadvantage even among patients with identical BCLC-C stage, and the results of multivariate analysis also suggested that impaired liver function was an independent predictor of poorer survival. Deteriorated liver function likely leads to early treatment discontinuation or limits tolerance to subsequent therapies (21). Moreover, hepatic decompensation may contribute more to mortality than tumor progression itself (22). These findings suggest that sintilimab-bevacizumab achieves meaningful antitumor activity in both CP-A and CP-B patients, while long-term prognosis remains heavily influenced by hepatic functional reserve.

Treatment of HCC in patients with impaired hepatic function continues to present a significant clinical challenge (23). Previous studies have reported median overall survival (mOS) of only 2–5 months in untreated patients with Child-Pugh B liver function (24, 25). Even with monotherapy, efficacy remains limited: sorafenib has shown survival of 2.5-5.2 months, lenvatinib demonstrates an mOS of around 3.7 months, while nivolumab yields a median PFS of 2.7 months and an ORR of 12% (26–29).

Most clinical trials have excluded or included only a limited number of CP-B patients, leaving a gap in evidence for this subgroup. In a previous multicenter retrospective study, it was reported that the mOS of HCC patients treated with atezolizumab combined with bevacizumab was 16.8 months and 6.7 months, respectively, for Child-Pugh A and Child-Pugh B patients, while in another multicenter retrospective study in Korea, the mOS of Child-Pugh B HCC patients was 7.7 months (30, 31). In an American study, 226 patients (70.1%) with Child-Pugh A and 86 patients (26.7%) with Child-Pugh B HCC were treated with atezolizumab and bevacizumab as first-line therapy, with a reported mOS of 21.6 months and 6.4 months in Child-Pugh A and Child-Pugh B patients (32). By contrast, our study observed a longer mOS of 15 months in CP-B patients, which may be partially explained by a lower proportion of patients with extrahepatic disease and by the optimized supportive and hepatic management in our cohort.

Importantly, the relatively favorable outcomes observed with sintilimab-bevacizumab in CP-B patients compared with other immune-based regimens may reflect both pharmacologic and clinical advantages. Pharmacologically, sintilimab and bevacizumab undergo proteolytic degradation rather than hepatic or renal metabolism, minimizing additional hepatic metabolic burden (33–36). This pharmacokinetic property may allow better drug exposure and tolerance in cirrhotic patients, particularly those with reduced hepatic reserve (37). Bevacizumab has been reported to modulate hepatic hemodynamics and alleviate vascular abnormalities, which may improve hepatic microcirculation and portal pressure, thereby facilitating hepatic perfusion and immune delivery (38, 39). Moreover, sintilimab’s distinct PD-1 binding characteristics may promote stable immune activation, potentially contributing to its sustained efficacy even in CP-B patients (40).

Our findings were consistent with the preliminary results presented at the 2024 ESMO Congress, where the mOS and mPFS in Child-Pugh B patients receiving FOLFOX-HAIC plus sintilimab and IBI305 were reported as 13.55 and 7.35 months, respectively (41). Among Child-Pugh A patients specifically, our observed mOS of 22 months is comparable to the results from CHANCE001 (19.2 months) and CHANCE2201 (22.5 months) (41, 42), supporting the robustness of this combination regimen even in broader real-world populations.

Given the inherent subjectivity of the Child-Pugh classification, we additionally incorporated the ALBI grade as a complementary tool for liver function assessment. Crucially, we identified on-treatment ALBI dynamics as a pivotal prognostic determinant. Patients who maintained or improved their ALBI grade at 6 months achieved superior OS (22 vs 17 months, p=0.027) and PFS (14 vs 8 months, p=0.014), indicating that early hepatic function stabilization, rather than baseline status alone, dictates long-term outcomes with immunotherapy combinations. Both univariate and multivariate analyses confirmed ALBI deterioration at 6 months as an independent predictor of poorer survival. These findings highlight its potential utility in refining patient stratification and treatment planning.

In this study, irAEs were defined as clinically confirmed immune-mediated events requiring immunosuppressive therapy or specialist management. Laboratory abnormalities such as elevated AST, ALT, or bilirubin were recorded separately and not classified as irAEs unless accompanied by clinical signs of immune-mediated hepatitis. This stricter definition likely contributed to the lower observed incidence (2.7%).

Safety remains a crucial concern in combined immunotherapy for HCC, particularly in cirrhotic patients. In our study, CP-B patients experienced a slightly higher overall AE rate (96.2% vs 90.4%) and a similar incidence of grade 3–5 events (34.6% vs 34.2%). Hypoalbuminemia was the most frequent AE in both groups, while thrombocytopenia predominated among severe AEs. CP-B patients had notably higher rates of gastrointestinal bleeding and hypoalbuminemia. Nonetheless, most AEs were grade 1–2 and manageable with supportive care. Serious AEs necessitating treatment discontinuation were uncommon and most frequently related to bleeding from portal hypertension.

We employed a comprehensive approach to AE risk mitigation: all cirrhotic patients underwent pre-treatment oesophagogastroduodenoscopy to screen for varices and received appropriate pharmacological or endoscopic intervention where indicated. Proton pump inhibitors were administered for ulcer prophylaxis, and transjugular intrahepatic portosystemic shunt (TIPS) was considered in select cases. Regular monitoring of coagulation parameters and occult bleeding, as well as prompt symptomatic management, ensured treatment safety. Permanent discontinuation was reserved for grade ≥3 hemorrhagic events.

Notably, sintilimab and bevacizumab are degraded by proteolytic enzymes rather than metabolized through hepatic or renal pathways, which likely contributes to their favorable hepatic and renal safety profiles (43). In our study, treatment-related liver and kidney function impairment was uncommon, particularly compared with reports of TKI-based regimens such as lenvatinib or sorafenib (29). This metabolic characteristic provides an additional safety advantage in patients with impaired hepatic reserve.

These findings emphasize the importance of integrated oncologic and hepatologic management in HCC patients, where both tumor burden and hepatic functional reserve dictate prognosis (44). CP-B status and ALBI deterioration independently predicted shorter OS, PFS, and time-to-discontinuation (TTD), reinforcing the need for individualized treatment planning and vigilant monitoring.

This study has several limitations. Its retrospective design introduces inherent selection bias, as reflected by baseline imbalances between groups, specifically the exclusion of 15 patients for “incomplete information” increases the possibility of such bias. Despite involving four institutions, the sample size remains relatively small, and the follow-up period was not long enough to evaluate long-term outcomes, as many patients were still being followed at the time of data cutoff. Moreover, all participants were enrolled from tertiary centers in China, and most had HBV-related HCC, which may limit the generalizability of our findings to populations with different etiologies or from other geographic regions.

In conclusion, the combination of sintilimab and bevacizumab demonstrates clinically meaningful antitumor activity in unresectable hepatocellular carcinoma across Child-Pugh A and B cohorts. However, survival outcomes were strongly affected by differences in hepatic function reserve and on-treatment liver functional dynamics. Although the regimen was generally well tolerated, patients with impaired liver reserve require vigilant monitoring and comprehensive supportive strategies to maximize therapeutic outcomes.

## Data Availability

The raw data supporting the conclusions of this article will be made available by the authors, without undue reservation.
